# Validation of suitable reference microRNAs for qRT-PCR in *Osmanthus fragrans* under abiotic stress, hormone and metal ion treatments

**DOI:** 10.3389/fpls.2025.1517225

**Published:** 2025-02-14

**Authors:** Yingting Zhang, Qingyu Yan, Hui Xia, Jie Yang, Xiangling Zeng, Zeqing Li, Xuan Cai, Jingjing Zou, Hongguo Chen

**Affiliations:** ^1^ National Forestry and Grassland Administration Engineering Research Center for Osmanthus fragrans, Hubei University of Science and Technology, Xianning, China; ^2^ Osmanthus Innovation Center of National Engineering Research Center for Floriculture, Hubei University of Science and Technology, Xianning, China; ^3^ College of Forestry, Central South University of Forestry and Technology, Changsha, China; ^4^ Research Center for Osmanthus fragrans, Xianning Research Academy of Industrial Technology of Osmanthus fragrans, Xianning, China

**Keywords:** *Osmanthus fragrans*, reference miRNAs, abiotic stress, hormone treatments, metal ion treatments

## Abstract

**Introduction:**

Sweet osmanthus (*Osmanthus fragrans*) is a prominent woody ornamental plant extensively utilized in horticulture, the food industry, cosmetics, and traditional Chinese medicine. MicroRNAs (miRNAs) are crucial regulators of gene regulation, playing a vital role in enabling plants to adapt to environmental fluctuations. Despite their significance, research on miRNA expression in *O. fragrans* under adverse stress conditions remains limited. Therefore, the selection of appropriate reference miRNAs is essential to ensure accurate miRNA expression analysis.

**Methods:**

In this study, qRT-PCR technology was combined with four algorithms (i.e., delta-Ct, geNorm, NormFinder, and BestKeeper) to systematically evaluate the expression stability of 14 candidate miRNAs across eleven environmental conditions, including under abiotic stress, under hormone and metal ion treatments, during flower opening and senescence, and across various tissues.

**Results:**

The results revealed that under hormone treatments, ofr-miR159b-3p, novel8, and novel3 exhibited high expression stability; under abiotic stress, ofr-miR159b-3p, novel8, ofr-miR403-3p, and novel2 demonstrated considerable stability; during metal ion treatments, novel3, ofr-miR159b-3p, novel33, novel2, and ofr-miR395e were identified as stable miRNAs; in different tissues, novel2 and ofr-miR395e were relatively stable; and during flower opening and senescence, novel33 and ofr-miR395e maintained stable expression.

**Discussion:**

This study represents the first comprehensive assessment of reference miRNA stability in *O. fragrans*, providing a reliable framework for miRNA expression analysis under diverse conditions, including flower development and senescence, abiotic stress, hormone treatments, and metal ion treatments. These findings carry significant implications for future research into the function of miRNAs.

## 1 Introduction

As one of China’s ten traditional famous flowers, sweet osmanthus (*Osmanthus fragrans*) is highly esteemed for its rich fragrance and attractive blossoms, making it significant in landscaping, food flavoring, cosmetics, and traditional Chinese medicine (Fu et al., 2022). However, with the intensification of global climate change and environmental pollution, *O. fragrans* encounters various environmental challenges during its growth, including drought, salinity, high temperatures, cold stress (Li et al., 2020; Zhu et al., 2022), and heavy metal contamination (Dang et al., 2022, 2024). These adverse conditions can disrupt the normal growth and development of *O. fragrans*, leading to a decline in flowering quality and potentially causing physiological disorders that may result in plant death under severe conditions. Therefore, studying the molecular response mechanisms of *O. fragrans* to these unfavorable conditions, particularly the regulation of gene expression, is crucial for enhancing its stress resistance and promoting its industrial applications.

MicroRNAs (miRNAs) have garnered significant attention in plant biology due to their critical role in regulating gene expression (Achkar et al., 2016; Millar, 2020). MiRNAs are a class of small, endogenous non-coding RNAs, typically 21–24 nucleotides in length. They regulate target gene expression by binding to the 3’ untranslated region (3’UTR) of target mRNAs, leading to mRNA degradation or inhibition of translation (Michlewski and Cáceres, 2018; Correia De Sousa et al., 2019). In plants, miRNAs are widely recognized as key regulators involved in various processes, including plant growth and development (Sun et al., 2018; Singh et al., 2020; Zhang et al., 2021b), hormone signaling (Long et al., 2017; Lian et al., 2018; Liu et al., 2020), and responses to environmental stress (Long et al., 2017; Sun et al., 2018; Dong et al., 2020; Singh et al., 2020; Ma and Hu, 2023). For instance, during the growth and development of tea oil camellia (*Camellia oleifera*) fruit, col-miR156, col-miR390, and col-miR395 differentially regulate genes associated with carbohydrate accumulation, while col-miR477 plays a crucial role in fatty acid synthesis (Liu et al., 2019). Furthermore, overexpression of gma-miR398c in *Arabidopsis* and soybean (*Glycine max*) has been shown to reduce drought resistance by negatively regulating peroxisome-related genes such as *GmCCS*, *GmCSD1a/b*, and *GmCSD2a/b/c* (Zhou et al., 2020). Similarly, osa-miR166 has been identified as essential for cadmium (Cd) accumulation and tolerance in rice (*Oryza sativa*) through the regulation of its target gene *OsHB4* (Ding et al., 2018). These studies underscore the critical role of miRNAs in regulating plant development and enhancing stress resistance. Although substantial advancements in miRNA research within plant biology, studies on miRNAs in *O. fragrans*, a prominent ornamental species, remain relatively limited. Current research has predominantly focused on miRNA expression and function in *O. fragrans* under normal growth conditions (Shi et al., 2021), with few investigations exploring their expression patterns under adverse conditions. Given that *O. fragrans* may regulate various genes through miRNAs in response to environmental stress, thereby enhancing its stress tolerance, it is crucial to systematically investigate the expression stability of miRNAs under these conditions. This is essential for advancing our understanding of the molecular mechanisms underlying its stress responses.

In gene expression analysis, selecting appropriate reference genes (RGs) for data normalization is crucial. Quantitative reverse transcription-polymerase chain reaction (qRT-PCR) is widely recognized for its sensitivity, specificity, speed, and high throughput, making it the preferred method for studying miRNA expression (Nolan et al., 2006; Vanguilder et al., 2008; Derveaux et al., 2010). However, the accuracy of qRT-PCR can be influenced by various factors, including RNA sample quality, reverse transcription efficiency, and the quality and quantity of cDNA (Derveaux et al., 2010). To minimize bias in qRT-PCR analysis, it is essential to validate suitable RGs across diverse experimental conditions, tissues, and species. Importantly, no universal RG exhibits stable expression across all experimental conditions. Therefore, it is necessary to screen and validate the most appropriate RGs for different species, tissues, or specific treatment conditions. Currently, commonly used RGs for miRNA expression analysis include short genes, such as 5.8S ribosomal RNA (*5.8S*) (Zhang et al., 2021a), *U6* small nuclear RNA (*U6*) (Duan et al., 2018), *18S* (Liu et al., 2023; He et al., 2024), actin (*ACT*) (Liu et al., 2023; Zhou et al., 2023), elongation factor-1α (*EF1B*) (Liu et al., 2023; He et al., 2024), glycerol-3-phosphate dehydrogenase (*GAPDH*) (Liu et al., 2023), and ubiquitin (*UBQ*) (Zhou et al., 2023; He et al., 2024). These RGs are widely employed in gene expression analysis due to their relatively stable expression across different experimental conditions. However, increasing evidence suggests that these traditional RGs may exhibit limitations regarding their stability and suitability for miRNA expression analysis. For instance, studies have indicated that RGs derived from conserved or novel miRNAs, such as *U6*, *5S* and *5.8S*, may be more stable than traditional protein-coding genes (Benz et al., 2013; Davoren et al., 2008). As research advances, suitable internal RGs for miRNA expression analysis have been identified in various species. For example, researchers have successfully screened and validated appropriate miRNA RGs in plants such as European aspen (*Populus tremula*) (Tang et al., 2019), Henry’s Lily (*Lilium henryi*) (Zhang et al., 2017; Jin et al., 2024) and Chinese cedar (*Cryptomeria fortunei*) (Zhang et al., 2021c, d). These studies provide valuable insights for selecting stable RGs under different experimental conditions. However, to date, no systematic evaluation of RGs for miRNA studies in *O. fragrans* has been reported.

To address this gap, the study utilized high-throughput sequencing to analyze miRNAs during the stages of flower opening and senescence in *Osmanthus fragrans*. From the sequencing data, nine miRNAs with high expression levels and stability were selected as candidate internal RGs, alongside five commonly used genes as additional candidates. The expression stability of these miRNAs was systematically evaluated using qRT-PCR across a range of experimental conditions, including abiotic stresses (low temperature, drought, and salinity), hormone treatments (abscisic acid (ABA), methyl jasmonate (MeJA), and ethephon), metal ion stresses (Fe²^+^, Al³^+^, and Cu²^+^), different tissues (root, seed, leaf, and flower), and during flower opening and senescence. The stability of these candidate RGs was evaluated using multiple methods, including delta-Ct, geNorm, NormFinder, BestKeeper, and RefFinder. These analyses identified suitable internal RGs, providing a scientific foundation and technical support for miRNA expression analysis and functional research in *O. fragrans*. This study not only provides scientific evidence and technical support for miRNA expression analysis in *O. fragrans*, but also offers valuable insights for the study of gene regulatory mechanisms in other economic crops and ornamental plants facing environmental stress.

## 2 Materials and methods

### 2.1 Plant material

A disease-free and vigorous “Chang’e” *O. fragrans* tree from Xianning (Hubei, China) was selected as the mother tree. In May 2023, semi-lignified branches measuring 12–16 cm, each with 2–3 lateral buds, were harvested as cuttings. The cuttings were first soaked in distilled water for 12 hours, then disinfected with 1% calcium hypochlorite for 10 minutes, and subsequently rinsed three times with distilled water. Following disinfection, the cuttings were immersed in a 0.1 g L^–1^ GGR rooting powder solution for 4 hours. They were then transplanted into a mixed soil matrix composed of peat, perlite, vermiculite, and yellow sand in a 1:1:1:1 ratio. The cuttings were cultivated at the *O. fragrans* base in Xianning.

Samples of *O. fragrans* were collected from various tissues, including roots, leaves, seeds, and flowers. A healthy and pest-free *O. fragrans* plant from Huazhong Agricultural University (114°21’W, 30°29’N) was selected for the study. Flower tissues were harvested at six developmental stages: S1 (stalk stage), S2 (early flowering stage), S3 (mid-flowering stage), S4 (full flowering stage), S5 (late flowering stage), and S6 (petal shedding stage) (Chen et al., 2021). Samples were collected at 10:00 am, with each stage represented by three biological replicates. The samples were rapidly frozen in liquid nitrogen and stored at –80°C for subsequent analysis.

### 2.2 Treatment of experimental materials

In March 2024, healthy and uniformly growing *O. fragrans* plants from the campus of Hubei University of Science and Technology (114°19’52’’E, 29°51’19’’N) were subjected to acclimation culture. In April 2024, 81 plants demonstrating uniform growth were selected for nine different stress treatments. For abiotic stress treatments, the plants were exposed to 4°C to simulate cold stress, and treated with 300 mM NaCl and 20% PEG-6000 to induce salt and drought stress, respectively (Zhang et al., 2021c, d). For hormone treatments, the plants were sprayed with 300 μM ABA, 300 μM MeJA, and 5 mM ethephon, respectively (Zhang et al., 2021c, d). Metal ion treatments were induced by spraying the cuttings with 3 mM CuSO_4_·5H_2_O, 3 mM AlCl_3_·6H_2_O, and 3 mM FeSO_4_, respectively (Khalid et al., 2020; Huang et al., 2021; Soares et al., 2022). Except for the cold stress treatment, each plant received 200 mL of the respective treatment solution, ensuring that all leaves were thoroughly wetted. The plants were then cultured in a light-controlled growth chamber at 25°C with a 12-hour light/12-hour dark cycle and 60% humidity. Three plants were used for each treatment, with three independent biological replicates (3 × 3 plants). Samples were collected at 0, 3, 6, 12, 24, and 72 hours post-treatment (Zhang et al., 2021c, d) and stored at –80°C for further analysis.

### 2.3 Identification of candidate RG and primer design

Common RGs such as *18S*, *ACT11*, α-tubulin 5 (*TUA5*), *U6*, and *UBQ4* previously identified in other species were locally blasted against the whole genome data of *O. fragrans* to identify candidate RGs with high homology. qRT-PCR primers were designed using Primer Premier 5.0 (Premier Biosoft International, Palo Alto, CA, USA), adhering to the following parameters: a PCR product length of 80–250 bp, a melting temperature (T_m_) of 58–62°C, and a GC content of 40–60%. Primer specificity was subsequently evaluated using e-PCR (https://yanglab.hzau.edu.cn/OfIR/tools/epcr/).

Based on small RNA-seq data, nine miRNAs with relatively high expression levels and fold changes < 1.4 were selected as candidates for RGs. These include five miRNAs (ofr-miR159b-3p, ofr-miR168b-5p, ofr-miR171a-3p, ofr-miR395e, and ofr-miR403-3p), as well as four novel miRNAs (novel2, novel3, novel8, and novel33) (**Supplementary Table S1**). In the miRNA sequences, uracil (U) was replaced with thymine (T), and modifications were made to achieve a T_m_ of 63–65°C, either by adding “G/C” bases at the 5’ end or deleting bases at the 5’/3’ ends. Reverse primers were provided by the miRcute miRNA qPCR Detection Kit (SYBR Green) (TianGen Biotech, Beijing, China), while the remaining primers (**Table 1**) were synthesized by Tsingke Biotech Co., Ltd. (Nanjing, Jiangsu Province, China).

**Table 1:** Gene sequence and primer information.

	Gene Sequence	Primer Sequence (5’–3’)	Amplicon Length (bp)	E (%)	R^2^
miR159b-3p	CUUUGGAUUGAAGGGAGCUCU	gcgcgcCTTTGGATTGAAGGGAGCTCT	80–150	92.987	0.994
miR168b-5p	UCGCUUGGUGCAGGUCGGGAA	ccgcTCGCTTGGTGCAGGTCGGGAA		98.711	0.984
miR171a-3p	UUGAGCCGCGCCAAUAUCACU	cgccgTTGAGCCGCGCCAATATCACT		97.919	0.996
miR395e	CUGAAGUGUUUGGGGGAACUC	ccggcCTGAAGTGTTTGGGGGAACTC		95.268	0.984
miR403-3p	UUAGAUUCACGCACAAACUCG	cgccgccgTTAGATTCACGCACAAACTCG		109.788	0.976
novel2	UUUCCUAUACCUCCCAUACCGA	ccgccgTTTCCTATACCTCCCATACCGA		104.177	0.987
novel3	UCAAGAUUGGGCAAUGAACCA	gcggcggTCAAGATTGGGCAATGAACCA		101.856	0.986
novel8	UUUCCUAUUCCUCCCAUACCGA	gcgccgTTTCCTATTCCTCCCATACCGA		111.362	0.983
novel33	UUGAACUCGUAUGCGAGCGCA	ccgcgTTGAACTCGTATGCGAGCGCA		100.614	0.993
*18S*	Chr10:28859089–28860895	CCATAAACGATGCCGACCAG	108	91.356	0.999
		GCCTTGCGACCATACTCCC			
*ACT11*	LYG009896	TCAATGATCGGAATGGAAGC	132	96.317	0.993
		ACCTGGGAACATGGTAGAACC			
*TUA5*	LYG023230	ATCATCGCTGACCACTTCTTTG	237	95.941	0.995
		GCCATGTATTTCCCGTGTCTT			
*U6*	Chr12:22899479–22899581	GGGGACATCCGATAAAATTG	87	95.241	0.982
		GGACCATTTCTCGATTTGTG			
*UBQ4*	LYG013775	ACTGCACCCTCCATTTGGT	165	103.178	0.999
		TGCCGTTCACGATTAGTTCTC			
Target genes					
miR166e-5p	GGAAUGUUGUCUGGCUCGAGA	ccgccGGAATGTTGTCTGGCTCGAGA	80–150	109.188	0.995
miR396b-3p	GUUCAAGAAAGCUGUGGGAGA	ccgccgGTTCAAGAAAGCTGTGGGAGA		102.004	1.000

E, amplification efficiency.

### 2.4 Extraction of total RNA and synthesis of first strand cDNA

Total RNA was extracted from each sample using the HiPure Plant RNA Mini Kit (Magen Biotechnology, Guangzhou, Guangdong, China), following the manufacturer’s instructions. RNA integrity was evaluated using 1% agarose gel electrophoresis, and concentration was determined using a NanoDrop 2000 spectrophotometer (Thermo Scientific, Wilmington, DE, USA). RNA samples that met the quality criteria were then used for cDNA synthesis. Specifically, 0.8 µg of RNA was reverse transcribed into cDNA using the miRcute miRNA First-Strand cDNA Synthesis Kit (Tiangen Biotech). The resulting cDNA was stored at –20°C.

### 2.5 Testing of primer specificity and E values

Accurately measured 2 μL of cDNA from each sample were combined to assess primer specificity through RT-PCR amplification using the Fast PCR Kit (Vazyme). The reaction mixture comprised 1.2 μL each of forward and reverse primers (10 μM), 1.2 μL of cDNA, 10 μL of 2× Rapid Taq Master Mix, and 6.4 μL of ddH_2_O. The amplification protocol involved an initial denaturation at 95°C for 3 minutes, followed by 35 cycles of denaturation at 95°C for 15 seconds, annealing at 59°C for 15 seconds, and extension at 72°C for 15 seconds, with a final extension at 72°C for 5 minutes. Primer specificity was subsequently verified using 2.0% (*w*/*v*) agarose gel electrophoresis.

The cDNA template was serially diluted in 5-fold increments (1:4, 1:24, 1:124, 1:624, 1:3124; cDNA:water, *v*:*v*). qRT-PCR was performed using a 20 μL reaction mixture, prepared according to the instructions of the miRcute Plus miRNA qPCR Detection Kit (SYBR Green). The reaction mixture comprised 10 μL of 2× miRcute Plus miRNA Premix (SYBR&ROX), 0.4 μL each of forward and reverse primers (10 μM), 2 μL of 10-fold diluted miRNA first-strand cDNA, and 7.2 μL of RNase-free ddH_2_O. The qRT-PCR was performed on the Tianlong Gentier 96E system (Tianlong Technology Co., Ltd., Xi’an, China) using the following program: an initial denaturation at 95°C for 15 minutes, followed by 40 cycles of denaturation at 94°C for 20 seconds, and annealing and extension at 60°C for 30 seconds. A melting curve analysis was performed from 60°C to 95°C to detect primer dimers and other amplification artifacts. Each gene assay included a non-template control, with reactions performed in triplicate biological replicates and triplicate technical replicates. Correlation coefficients (R^2^) and amplification efficiencies (E values) were calculated from the qRT-PCR data (Whistler et al., 2010).

### 2.6 Gene expression stability analysis

cDNA samples were diluted 10-fold (1:9, cDNA:water, *v*:*v*) and analyzed by qRT-PCR to obtain the raw Ct values. The expression stability of the RGs was assessed using the following algorithms: delta-Ct (Silver et al., 2006), geNorm (*v*3.5) (Vandesompele et al., 2002), NormFinder (*v*0.953) (Andersen et al., 2004), and BestKeeper (*v*1.0) (Pfaffl et al., 2004). Specifically, geNorm and NormFinder converted the Ct values to 2^–ΔCt^ (where delta-Ct = Ct value – minimum Ct value for each group) for stability assessment. BestKeeper evaluated stability based on the E values derived from the Ct values using the LinRegPCR program, considering the coefficient of variation (CV) and standard deviation (SD). Additionally, geNorm determined the optimal number of RGs by calculating the pairwise variation (V_n_/V_n+1_) between consecutive normalization factors.

The geometric mean ranking was calculated based on the average rankings of genes across various treatments, tissues, or all samples, as determined by the four algorithms. Additionally, the stability analysis results of the RGs were comprehensively validated using RefFinder (https://blooge.cn/RefFinder/) (Xie et al., 2023).

### 2.7 Validation of RGs using qRT-PCR

To evaluate the reliability of the selected RGs, we used the most stable RG combination and the least stable RG to normalize the expression levels of two miRNAs, ofr-miR166e-5p and ofr-miR396b-3p, under each experimental condition using qRT-PCR. Primers for these miRNAs were designed using the same method as for the candidate reference miRNAs, with the specific primers listed in **Table 1**. The relative expression levels of the miRNAs were calculated using the 2^–ΔΔCt^ method (Livak and Schmittgen, 2000).

## 3 Results

### 3.1 Primer specificity and amplification E values evaluation

**Figure 1:**
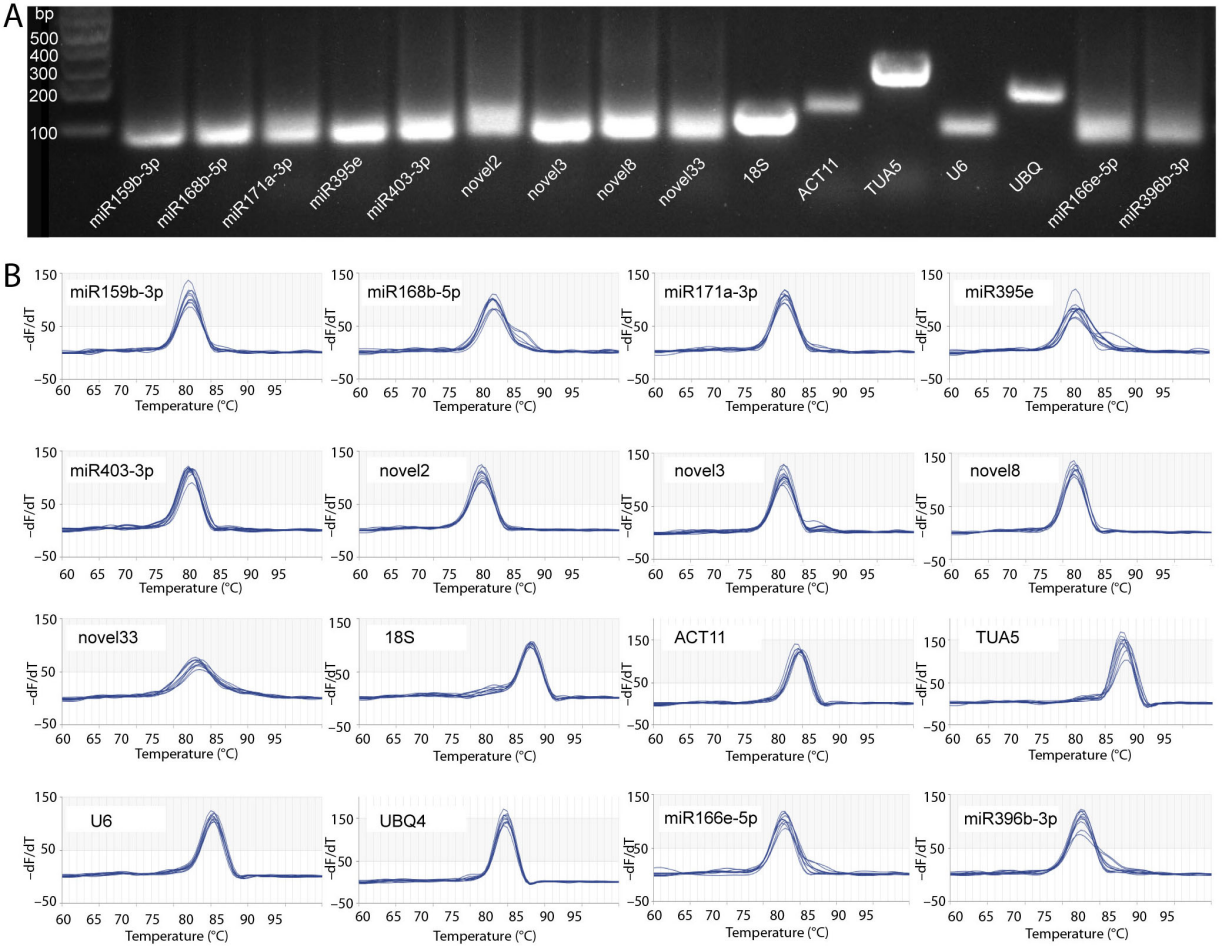
Validation of primer specificity. **(A)** Agarose gel electrophoresis. **(B)** Melting curves of qRT-PCR primers. The gene abbreviations are as follows: *18S*, 18S ribosomal RNA; *ACT11*, actin 11; *TUA5*, tubulin alpha 5; and *UBQ4*, ubiquitin 4.

In this study, a total of 14 candidate RGs were selected for gene normalization analysis, comprising 5 genes and 9 miRNAs (**Table 1**). The E values of the candidate RG primers ranged from 91.356% to 111.362%, with R2 values ≥ 0.976 (**Table 1**). Primer specificity was evaluated using agarose gel electrophoresis and melting curve analyses. The agarose gel electrophoresis results demonstrated that all primers successfully amplified PCR products of the expected size, confirming the correct design and specificity of the primers (**Figure 1A**). Melting curve analysis further verified that each primer produced a single melting peak (**Figure 1B**), suggesting that the amplified products were free from non-specific amplification or primer dimers.

### 3.2 Expression level analysis of candidate RGs

**Figure 2:**
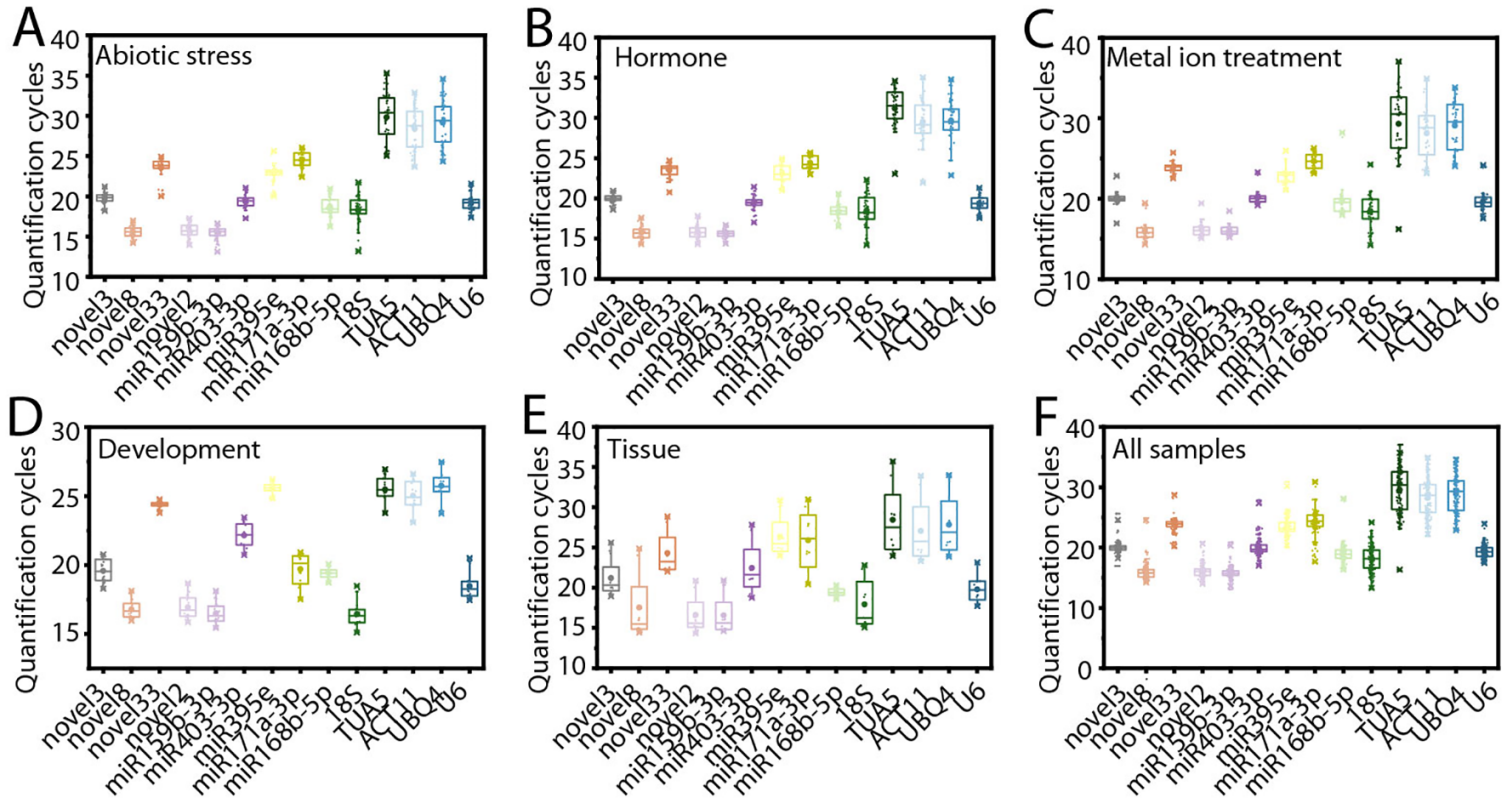
Variation in cycle threshold (Ct) values for the 14 candidate reference genes (RGs). **(A)** Abiotic stress. Plants were subjected to individual treatments, including cold, salt, and drought stress, and variation in Ct values are calculated based on data from these treatments. **(B)** Hormone treatments. Plants were treated with ABA, MeJA, and ethephon, and variation in Ct values are calculated based on data from these treatments. **(C)** Metal ion treatments. Plants were exposed to Al^3+^, Cu^2+^, and Fe^2+^ treatment, and variation in Ct values are calculated based on data from these treatments. **(D)** Flower opening and senescence. **(E)** Different tissues. **(F)** Across all samples. It is a collection of Ct values from all treatments, different tissues, and flowering stages.

To evaluate the suitability of the candidate RGs, their expression levels were analyzed across all samples using qRT-PCR. These samples included those exposed to various conditions: abiotic stress (low temperature, drought, and salt stress), hormone treatments (ABA, MeJA, and ethephon), metal ion treatments (Fe²^+^, Al³^+^, and Cu²^+^), different tissues (roots, leaves, seeds, and flowers), and during the flower opening and senescence (**Figure 2**).

Under abiotic stress, the average Ct values of the samples ranged from 15.472 (ofr-miR159b-3p) to 29.802 (*TUA5*) (**Figure 2A**). Notably, novel8, novel2, and ofr-miR159b-3p exhibited relatively low Ct values, indicating higher initial copy numbers and thus higher expression levels in the samples (Bustin et al., 2009). Among these, novel8 demonstrated the smallest variation in Ct values, with a range of only 2.772 (**Figure 2A**). Under hormone treatments, the average Ct values varied from 15.625 (ofr-miR159b-3p) to 31.180 (*TUA5*) (**Figure 2B**). Among these, ofr-miR159b-3p displayed the smallest variation in Ct values, with a range of just 2.304 (**Figure 2B**). For metal ion treatments, ofr-miR171a-3p exhibited the least variation in Ct values, with a range of 3.164 (**Figure 2C**). During flower opening and senescence, novel33 had the smallest Ct value variation (1.000) (**Figure 2D**). In different tissues, ofr-miR168b-5p showed the least variation in Ct values (1.718) (**Figure 2E**). Across all samples, *U6* had the smallest variation in Ct values, with a range of 6.758 (**Figure 2F**). These results highlight the relative stability of certain genes in their expression levels, suggesting their potential as stable RGs. However, further in-depth analysis is required to confirm their suitability.

### 3.3 Expression stability of candidate genes based on delta-Ct analysis

To evaluate the stability of the candidate RGs, we employed the delta-Ct method, which compares the relative expression levels of gene across various sample groups. The RGs were ranked according to the reproducibility of the average standard deviation (STDEV) of gene expression differences between samples (Silver et al., 2006). Generally, a lower STDEV value indicates greater stability in gene expression.

**Figure 3:**
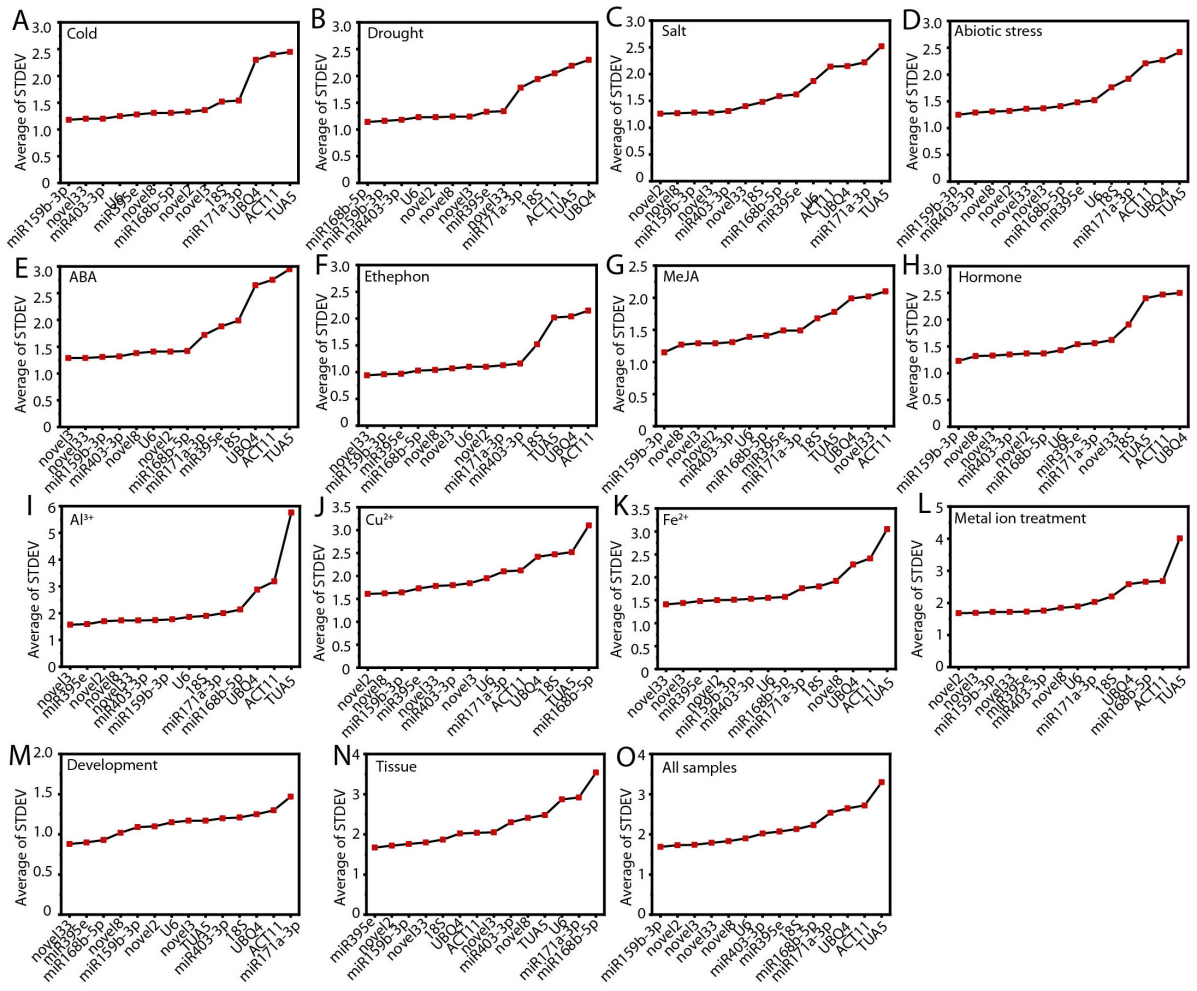
The average standard deviation (STDEV) obtained from the delta-Ct analysis across different conditions. **(A)** Cold, **(B)** salt, **(C)** drought, and **(D)** abiotic stress; **(E)** ABA, **(F)** MeJA, **(G)** ethephon, **(H)** hormone, **(I)** Al^3+^, **(J)** Cu^2+^, **(K)** Fe^2+^, and **(L)** metal ion treatments; **(M)** flowering stage; **(N)** different tissues; and **(O)** all samples.

Under low temperature stress, abiotic stress, MeJA treatment, hormone treatments, and across all samples, ofr-miR159b-3p exhibited the most stable expression (**Figures 3A, D, G, H, O**). Novel2 was more stable than other RGs under salt stress, Cu²^+^ treatment, and metal ion stress (**Figures 3C, J, L**). Novel3 was the most stable gene under ABA and Al³^+^ treatments (**Figures 3E, I**). Novel33 showed the highest stability during ethephon treatment, Fe²^+^ treatment, and throughout the flower opening and senescence stages (**Figures 3F, K, M**). Under drought stress and across different tissues, ofr-miR168b-5p and ofr-miR395e were identified as the most stable RGs, respectively (**Figures 3B, N**). Notably, *TUA5* (10, 66.67%) and *ACT11* (10, 66.67%) exhibited the least stability across most conditions (**Figure 3**; **Supplementary Table S2**).

### 3.4 Expression stability of candidate RGs based on geNorm analysis

**Figure 4:**
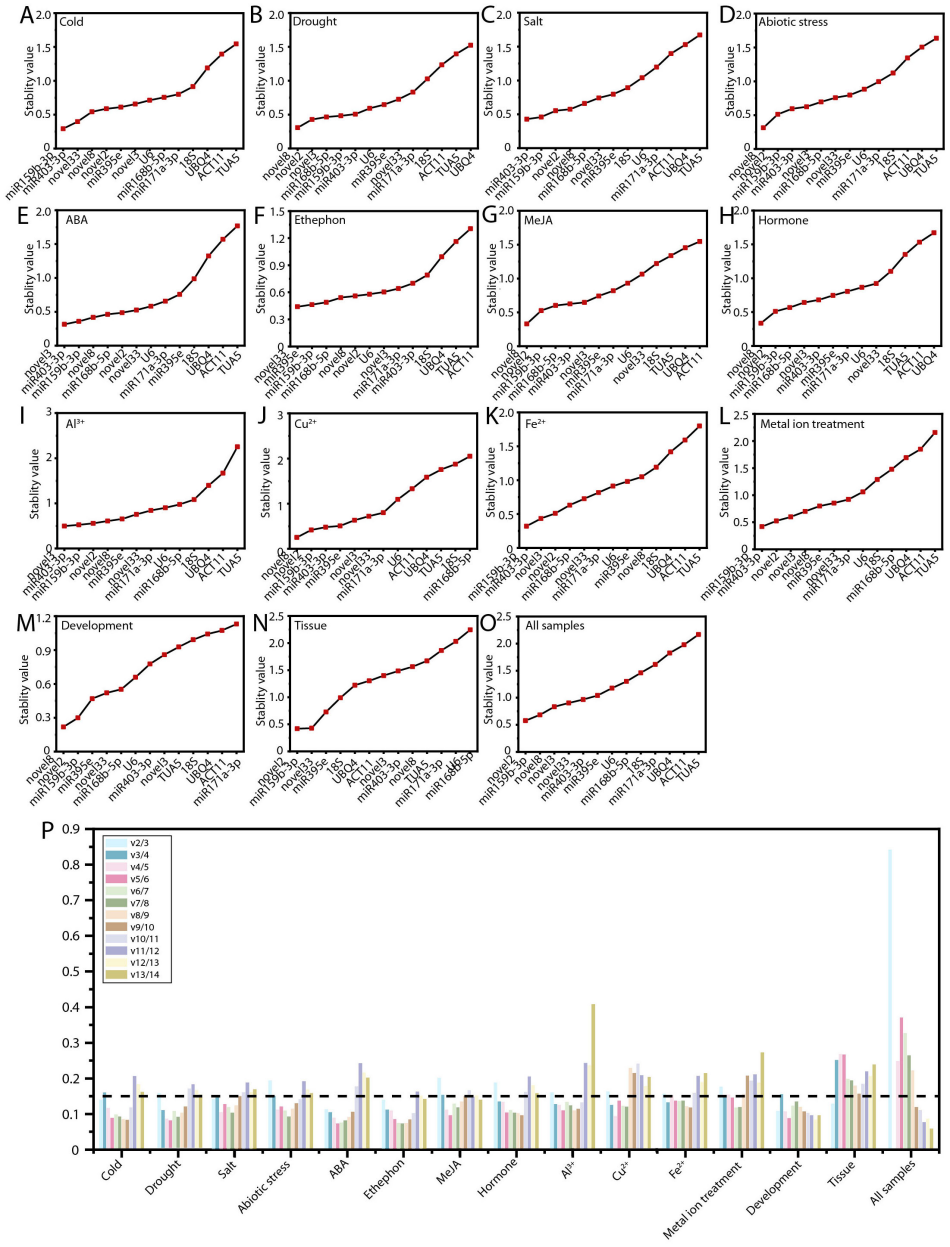
Expression stability of RGs as calculated by geNorm. Expression stability of RGs under the following experimental conditions: **(A)** Cold, **(B)** salt, **(C)** drought, and **(D)** abiotic stress; **(E)** ABA, **(F)** MeJA, **(G)** ethephon, **(H)** hormone, **(I)** Al^3+^, **(J)** Cu^2+^, **(K)** Fe^2+^, and **(L)** metal ion treatments; **(M)** flowering stage; **(N)** different tissues; and **(O)** all samples. **(P)** Determination of the optimal RG combinations.

The geNorm algorithm evaluates the stability of RGs by calculating the mean variation value, commonly known as the M value, where a lower M value indicates greater gene stability (Vandesompele et al., 2002). The results of the geNorm analysis are illustrated in **Figure 4**. ofr-miR159b-3p demonstrated the highest stability under cold stress, Fe²^+^ treatment, and metal ion stress (**Figures 4A, K, L**). Novel8 was identified as the most stable RG under drought stress, abiotic stress, MeJA treatment, hormone treatments, Cu²^+^ treatment, and during flower opening and senescence (**Figures 4B, D, G, H, J, M**). Novel3 exhibited the greatest stability under ABA and Al³^+^ treatments (**Figures 4E, I**). Across various tissues and all samples, novel2 displayed superior stability compared to other RGs (**Figures 4N, O**). Under salt stress and ethephon treatment, ofr-miR403-3p and novel33 were the most stable, respectively (**Figures 4C, F**). Notably, consistent with the delta-Ct analysis, *TUA5* (10, 66.67%) and *ACT11* (10, 66.67%) were the least stable across most conditions (**Figures 4A–O**; **Supplementary Table S2**).

A single RG is generally insufficient for achieving the stability required for accurate normalization. Therefore, it is essential to use two or more RGs to minimize error and ensure more reliable results. The geNorm algorithm determines the optimal number of candidate RGs by calculating the pairwise variation (V_n_/V_n+1_), with a threshold of 0.15 to identify the ideal number of RGs for normalization. For PEG treatment, hormone treatments, Fe²^+^ treatment, Al³^+^ treatment, and Cu²^+^ treatment, the V_3_/V_4_ value was below 0.15, indicating that a combination of three RGs is most appropriate (**Figure 4P**). For MeJA treatment and abiotic stress, the V_4_/V_5_ value was below 0.15, suggesting that the optimal RG combination includes four genes (**Figure 4P**). In metal ion treatments, the use of five RGs is necessary (**Figure 4P**). Across all samples, nine RGs are required for accurate normalization (**Figure 4P**).

### 3.5 Analysis of candidate gene expression stability based on NormFinder

**Figure 5:**
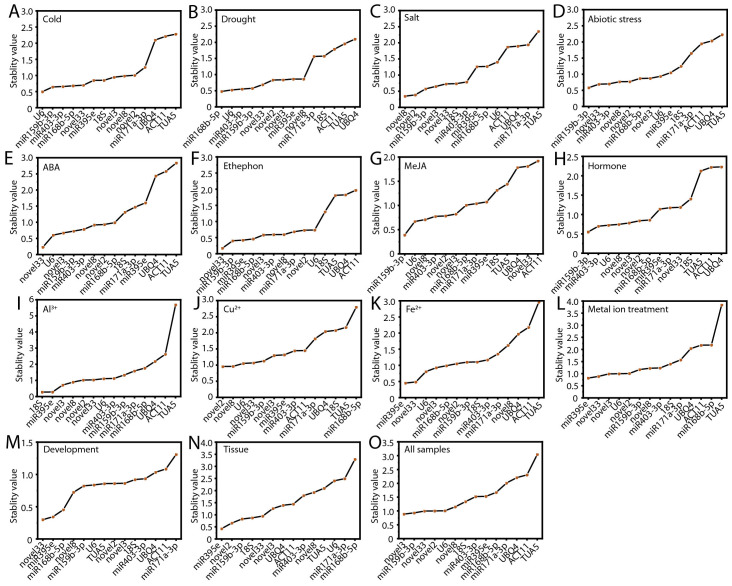
Evaluation of candidate RG expression stability using NormFinder. **(A)** Cold, **(B)** salt, **(C)** drought, and **(D)** abiotic stress; **(E)** ABA, **(F)** MeJA, **(G)** ethephon, **(H)** hormone, **(I)** Al^3+^, **(J)** Cu^2+^, **(K)** Fe^2+^, and **(L)** metal ion treatments; **(M)** flowering stages; **(N)** different tissues; and **(O)** across all samples.

NormFinder was employed to evaluate the expression stability of candidate RGs by ranking them according to variance analysis results (Andersen et al., 2004). A low stability value indicates that the gene exhibits significant variability under different experimental conditions, making it unsuitable as a RG. In contrast, a high stability value indicates that the gene shows minimal variation, thus demonstrating higher stability and being more reliable as a RG. The NormFinder analysis identified the most stable RGs across various experimental conditions, as depicted in **Figure 5**. Under cold, drought, and salt stress conditions, the most optimal RGs were *U6*, ofr-miR168b-5p, and novel8, respectively (**Figures 5A–C**). For Al³^+^ and Cu²^+^ treatments, as well as across all samples, *18S*, novel2, and novel3 exhibited the highest stability compared to other genes (**Figures 5I, J, O**). Similarly, under abiotic stress, MeJA treatment, and hormone treatments, ofr-miR159b-3p demonstrated the greatest stability (**Figures 5D, G, H**). Under ABA treatment, ethephon treatment, and the flower opening and senescence process, novel33 was identified as the most stable RG (**Figures 5E, F, M**). For Fe²^+^, metal ion treatments, and different tissues, ofr-miR395e emerged as the optimal RG (**Figures 5K, L, N**). It is noteworthy that, consistent with the delta-Ct and geNorm analyses, *TUA5* (10, 66.67%) and *ACT11* (9, 60.00%) exhibited the lowest stability across most conditions (**Figure 5**; **Supplementary Table S2**).

### 3.6 Evaluation of candidate RG expression stability using BestKeeper

**Figure 6:**
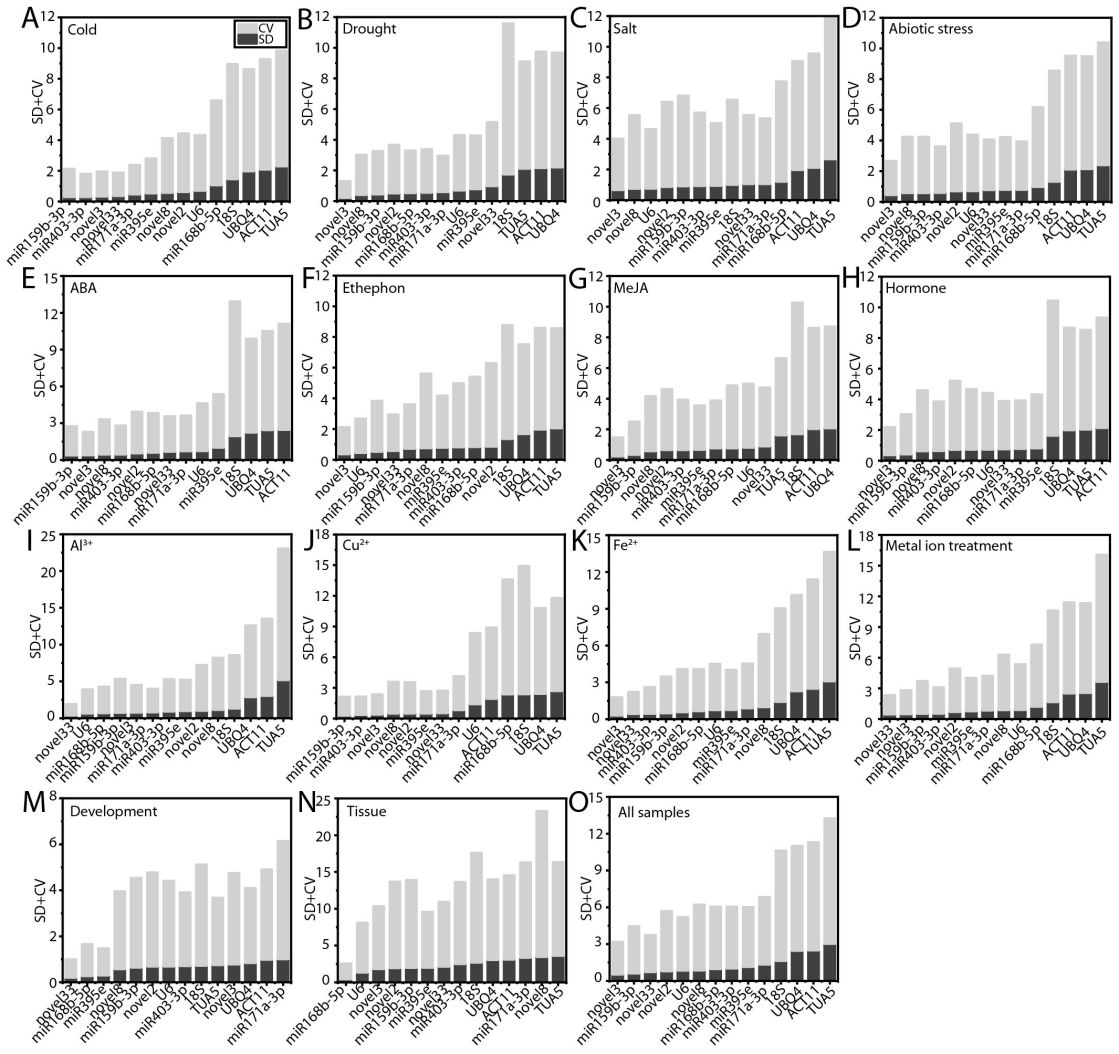
Evaluation of candidate RG stability using BestKeeper. **(A)** Cold, **(B)** salt, **(C)** drought, and **(D)** abiotic stress; **(E)** ABA, **(F)** MeJA, **(G)** ethephon, **(H)** hormone, **(I)** Al^3+^, **(J)** Cu^2+^, **(K)** Fe^2+^, and **(L)** metal ion treatments; **(M)** flowering stages; **(N)** different tissues; and **(O)** across all samples.

BestKeeper evaluates the stability of RGs by calculating the SD and CV of the Ct values (Pfaffl et al., 2004). RGs with an SD of less than 1.0 are considered to exhibit stable expression, as a lower CV generally indicates greater stability. Under cold stress, ABA treatment, and Cu²^+^ treatment, ofr-miR159b-3p was identified as the most stable RG (**Figures 6A, E, J**). Novel3 proved to be the optimal RG under drought stress, salt stress, abiotic stresses, ethephon treatment, MeJA treatment, hormone treatments, Fe²^+^ treatment, and across all samples (**Figures 6B–D, F–H, K, O**). Novel33 exhibited the highest stability during Al³^+^ treatment, metal ion treatments, and throughout the processes of flower opening and senescence (**Figures 6I, L, M**). Additionally, ofr-miR168b-5p displayed superior stability compared to other RGs across various tissues (**Figure 6N**). Consistent with the delta-Ct, geNorm, and NormFinder analyses, *TUA5* (12 instances, 75.00%) and *ACT11* (10 instances, 66.67%) showed the lowest stability under most conditions (**Figure 6**).

### 3.7 Evaluation of candidate gene stability using RefFinder

Different algorithms utilize distinct principles to evaluate gene stability, often resulting in variations in their rankings. To achieve a more comprehensive assessment, we calculated the geometric mean of stability scores across delta-Ct, geNorm, NormFinder, and BestKeeper for each experimental condition. The optimal RG combinations were subsequently selected based on geNorm analysis. The results were as follows: for ethephon treatment: novel33 and ofr-miR159b-3p; for ABA treatment: novel3 and ofr-miR159b-3p; for MeJA treatment: ofr-miR159b-3p, novel8, novel2, and novel3; for hormone treatments: ofr-miR159b-3p, novel8, and novel3; for drought stress: ofr-miR168b-5p, ofr-miR159b-3p, and novel2; for salt stress: novel8 and novel3; for cold stress: ofr-miR159b-3p and ofr-miR403-3p; under abiotic stress: ofr-miR159b-3p, novel8, ofr-miR403-3p, and novel2; for Fe²^+^ treatment: novel3, novel33, and ofr-miR159b-3p; for Al³^+^ treatment: novel3, ofr-miR395e, and novel33; for Cu²^+^ treatment: novel2, novel8, and ofr-miR159b-3p; for metal ion stress: novel3, ofr-miR159b-3p, novel2, novel33, and ofr-miR395e; across various tissues: novel2 and ofr-miR395e; during flowering stages: novel33 and ofr-miR395e; and across all samples: ofr-miR159b-3p, novel3, novel2, novel33, novel8, *U6*, ofr-miR403-3p, ofr-miR395e, and ofr-miR168b-5p (**Table 2**). Additionally, the least stable genes identified were as follows: *TUA5* for cold stress, salt stress, abiotic stress, ABA treatment, Al³^+^ treatment, Fe²^+^ treatment, metal ion stress, and across all samples; *UBQ4* for drought stress, and hormone treatments; *ACT11* for MeJA, and ethephon treatments; ofr-miR171a-3p for different tissues, and during flowering stages; and ofr-miR168b-5p for Cu²^+^ treatment (**Table 2**).

**Table 2:** The comprehensive ranking of RGs across various experimental conditions was determined by calculating the geometric mean of rankings from four different methods.

	Cold	Salt	Drought	Abiotic stress	ABA	MeJA	Ethephon	Hormone
1	miR159b-3p	novel3	miR168b-5p	miR159b-3p	novel3	miR159b-3p	novel33	miR159b-3p
2	miR403-3p	novel8	novel8	novel8	miR159b-3p	novel8	miR159b-3p	novel8
3	novel33	novel2	miR159b-3p	miR403-3p	miR403-3p	novel2	miR395e	novel3
4	*U6*	miR159b-3p	novel2	novel2	novel33	novel3	novel3	novel2
5	miR395e	miR403-3p	novel3	novel3	novel8	miR403-3p	miR168b-5p	miR403-3p
6	novel3	novel33	miR403-3p	novel33	*U6*	*U6*	*U6*	*U6*
7	novel8	*U6*	*U6*	miR168b-5p	novel2	miR168b-5p	novel8	miR168b-5p
8	miR168b-5p	*18S*	novel33	*U6*	miR168b-5p	miR395e	miR171a-3p	miR395e
9	novel2	miR395e	miR395e	miR395e	miR171a-3p	miR171a-3p	novel2	miR171a-3p
10	miR171a-3p	miR168b-5p	miR171a-3p	miR171a-3p	miR395e	*18S*	miR403-3p	novel33
11	*18S*	*ACT11*	*18S*	*18S*	*18S*	*TUA5*	*18S*	*18S*
12	*UBQ4*	miR171a-3p	*ACT11*	*ACT11*	*UBQ4*	novel33	*UBQ4*	*TUA5*
13	*ACT11*	*UBQ4*	*TUA5*	*UBQ4*	*ACT11*	*UBQ4*	*TUA5*	*ACT11*
14	*TUA5*	*TUA5*	*UBQ4*	*TUA5*	*TUA5*	*ACT11*	*ACT11*	*UBQ4*
	Al^3+^	Cu^2+^	Fe^2+^	Metal ion	Flowering stages	Different tissues	All samples	
1	novel3	novel2	novel33	novel3	novel33	novel2	miR159b-3p	
2	miR395e	novel8	novel3	miR159b-3p	miR395e	miR395e	novel3	
3	novel33	miR159b-3p	miR159b-3p	novel33	novel8	miR159b-3p	novel2	
4	miR403-3p	miR403-3p	miR403-3p	novel2	miR168b-5p	novel33	novel33	
5	novel2	novel3	miR395e	miR395e	novel2	*18S*	novel8	
6	miR159b-3p	miR395e	novel2	miR403-3p	miR159b-3p	novel3	*U6*	
7	novel8	novel33	*U6*	novel8	*U6*	*UBQ4*	miR403-3p	
8	*U6*	*U6*	miR168b-5p	*U6*	*TUA5*	miR168b-5p	miR395e	
9	*18S*	miR171a-3p	miR171a-3p	miR171a-3p	miR403-3p	*U6*	miR168b-5p	
10	miR168b-5p	*ACT11*	*18S*	*18S*	novel3	*ACT11*	*18S*	
11	miR171a-3p	*UBQ4*	novel8	miR168b-5p	*18S*	miR403-3p	miR171a-3p	
12	*UBQ4*	*18S*	*UBQ4*	*UBQ4*	*UBQ4*	novel8	*UBQ4*	
13	*ACT11*	*TUA5*	*ACT11*	*ACT11*	*ACT11*	*TUA5*	*ACT11*	
14	*TUA5*	miR168b-5p	*TUA5*	*TUA5*	miR171a-3p	miR171a-3p	*TUA5*	

The optimal RG combinations and the least stable genes identified by RefFinder are largely consistent with those obtained from the geometric mean analysis. The only exception is under drought stress, where the most stable RG combination was identified as ofr-miR168b-5p, ofr-miR159b-3p, and novel2 (rather than novel8) (**Table 2**; **Supplementary Table S3**).

### 3.8 Validation of RGs

**Figure 7:**
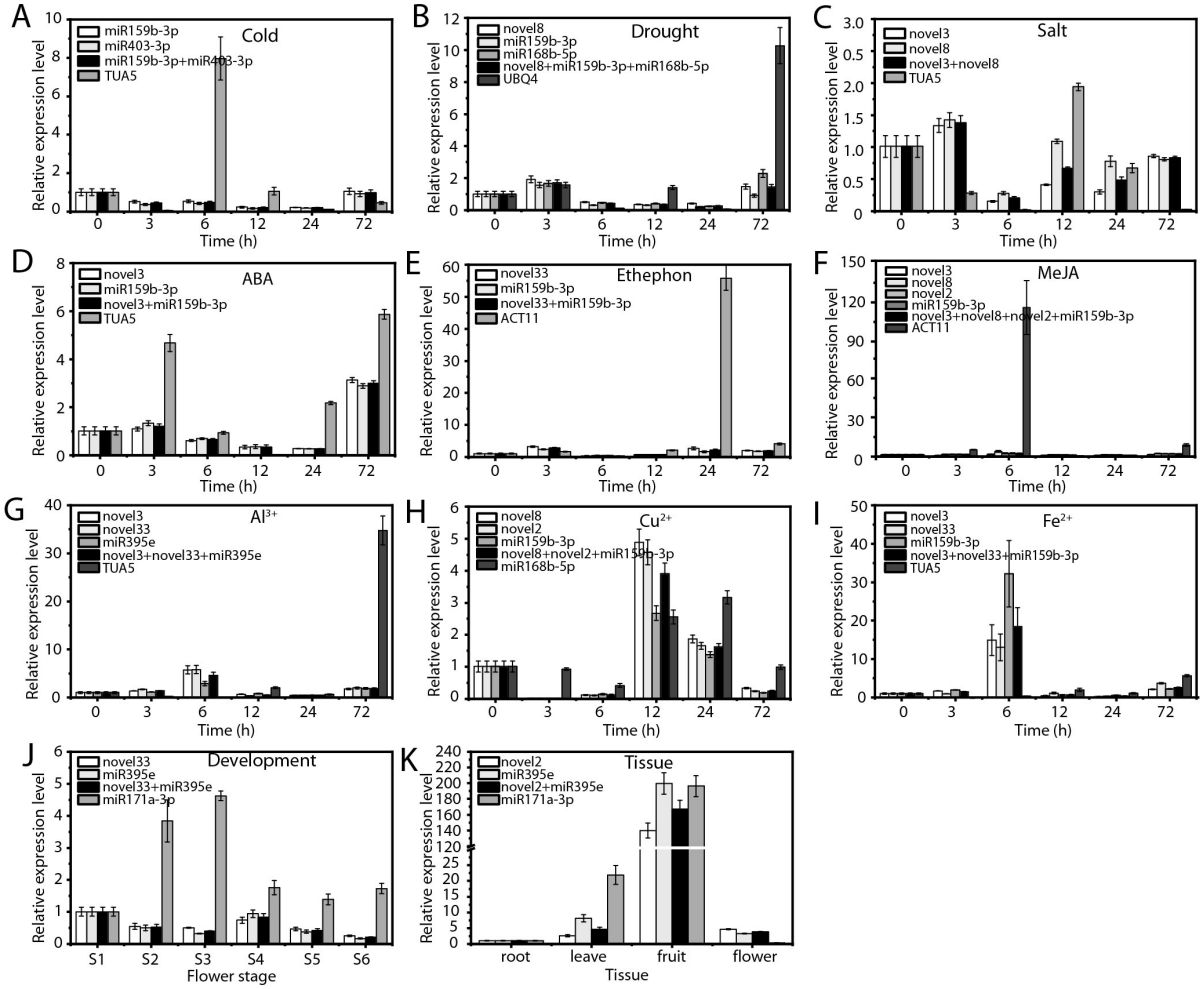
Relative expression levels of ofr-miR166e-5p. **(A)** Cold stress, **(B)** drought stress, and **(C)** salt stress; **(D)** ABA treatment, **(E)** ethephon treatment, **(F)** MeJA treatment, **(G)** Al³^+^ treatment, **(H)** Cu²^+^ treatment, and **(I)** Fe²^+^ treatment; **(J)** flowering stage; and **(K)** various tissues. The expression level ofr-miR166e-5p in each experimental condition was normalized using qRT-PCR with the most stable RG combination and the least stable RG, respectively.

**Figure 8:**
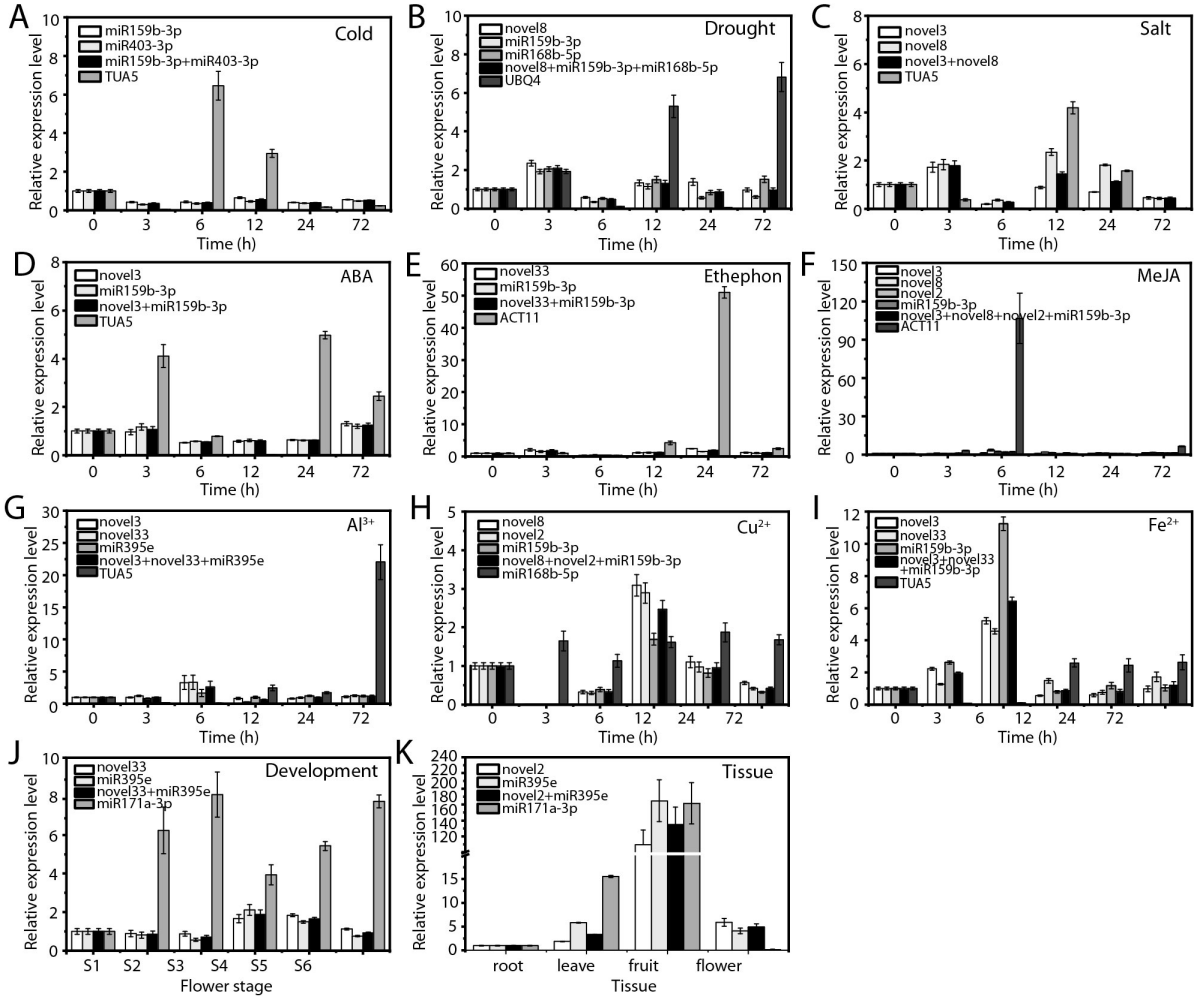
Relative expression levels of ofr-miR396b-3p. **(A)** Cold, **(B)** drought, and **(C)** salt stress; **(D)** ABA, **(E)** ethephon, **(F)** MeJA, **(G)** Al^3+^, **(H)** Cu^2+^, and **(I)** Fe^2+^ treatments; **(J)** flowering stage; and **(K)** various tissues. The expression level ofr-miR396b-3p in each experimental condition was normalized using qRT-PCR with the most stable RG combination and the least stable RG, respectively.

To ensure the accuracy of stable RG expression, we evaluated the relative expression levels of two target genes, ofr-miR166e-5p (**Figure 7**) and ofr-miR396b-3p (**Figure 8**), using both stable and unstable RG combinations under various experimental conditions.

When the most stable RGs were employed, the expression patterns and levels of the target genes remained consistent across various experimental conditions (**Figures 7**, **8**). In contrast, the use of unstable RGs resulted in significant discrepancies in the expression patterns of these target genes (**Figures 7**, **8**). For example, under cold stress, the use of stable RGs indicated that ofr-miR166e-5p and ofr-miR396b-3p exhibited higher expression levels at 0 and 72 hours (**Figures 7A**, **8A**), whereas the use of the unstable RG *TUA5* led to peak expression levels of these target genes at 6 hours (**Figures 7A**, **8A**). Similarly, under drought treatment, ofr-miR166e-5p and ofr-miR396b-3p reached their highest expression levels at 3 hours with stable RGs, while peak expression occurred at 12 hours when unstable RGs were used (**Figures 7C**, **8C**). Across various treatments, although the expression patterns of the target genes were generally similar when using different stable RGs, their expression levels varied between treatments (**Figures 7**, **8**). For instance, during flowering and senescence, stable RGs such as novel2 and ofr-miR395e, either individually or in combination, showed that the target genes had the highest expression in seeds, with lower levels in flowers and roots. In contrast, using the unstable RG ofr-miR171a-3p resulted in similar expression trends, but expression levels in flowers were only 14.43% and 3.32% of those observed with stable RGs (**Figures 7K**, **8K**). Overall, the selection of appropriate RGs is crucial for accurate normalization of target gene expression, as incorrect RG selection can lead to inaccurate estimates of relative expression levels.

## 4 Discussion

qRT-PCR technology is extensively used to identify appropriate RGs for normalizing gene expression (Zhu et al., 2020; Zhang et al., 2021c). Although several studies have identified miRNA RGs in various plant species, such as poplar (Tang et al., 2019), Chinese cedar (*Cryptomeria fortunei*) (Zhang et al., 2021c, d), and *Lilium* (Zhang et al., 2017), no such research has yet been reported for *O. fragrans*. This study presents a systematic evaluation of miRNA expression stability across a range of conditions, including abiotic stresses, hormone treatments, metal ion stresses, different tissues, and flower developmental stages in *O. fragrans.* It establishes a solid foundation for gene expression analysis under diverse environmental conditions and provides valuable insights into the stress resistance mechanisms of *O. fragrans*.

In this study, the E values of the candidate RG primers ranged from 91.356% to 111.362%, with R² values ≥ 0.976 (**Table 1**). These results underscore the high accuracy, efficiency, and sensitivity of the primers employed for RG screening, providing a reliable foundation for subsequent reference miRNA screening and expression analysis. Under various experimental conditions, including abiotic stresses, hormone treatments, and metal ion treatments, the Ct value variations for novel8, ofr-miR159b-3p, and ofr-miR171a-3p were minimal (**Figures 2A–C**). Similarly, during flower opening and senescence, across different tissues, and among all samples, Ct value variations for novel33, ofr-miR168b-5p, and *U6* were also minimal, respectively (**Figures 2D–F**). These findings align with previous studies conducted across a range of plant species, including poplar (Tang et al., 2019; Zhang et al., 2021a), *C. fortunei* (Zhang et al., 2021c, d), tomato (*Solanum lycopersicum*) (Løvdal and Lillo, 2009), soybean (Kulcheski et al., 2010; Liu et al., 2016), *Lilium* (Zhang et al., 2017; Jin et al., 2024), grapevine (*Vitis vinifera*) (Luo et al., 2018), and atlantic salmon (*Salmo salar*) (Johansen and Andreassen, 2014), which demonstrate that gene expression stability varies across different biological contexts. Therefore, the selection of appropriate RGs for specific experimental conditions is essential for accurate miRNA expression normalization.

**Figure 9:**
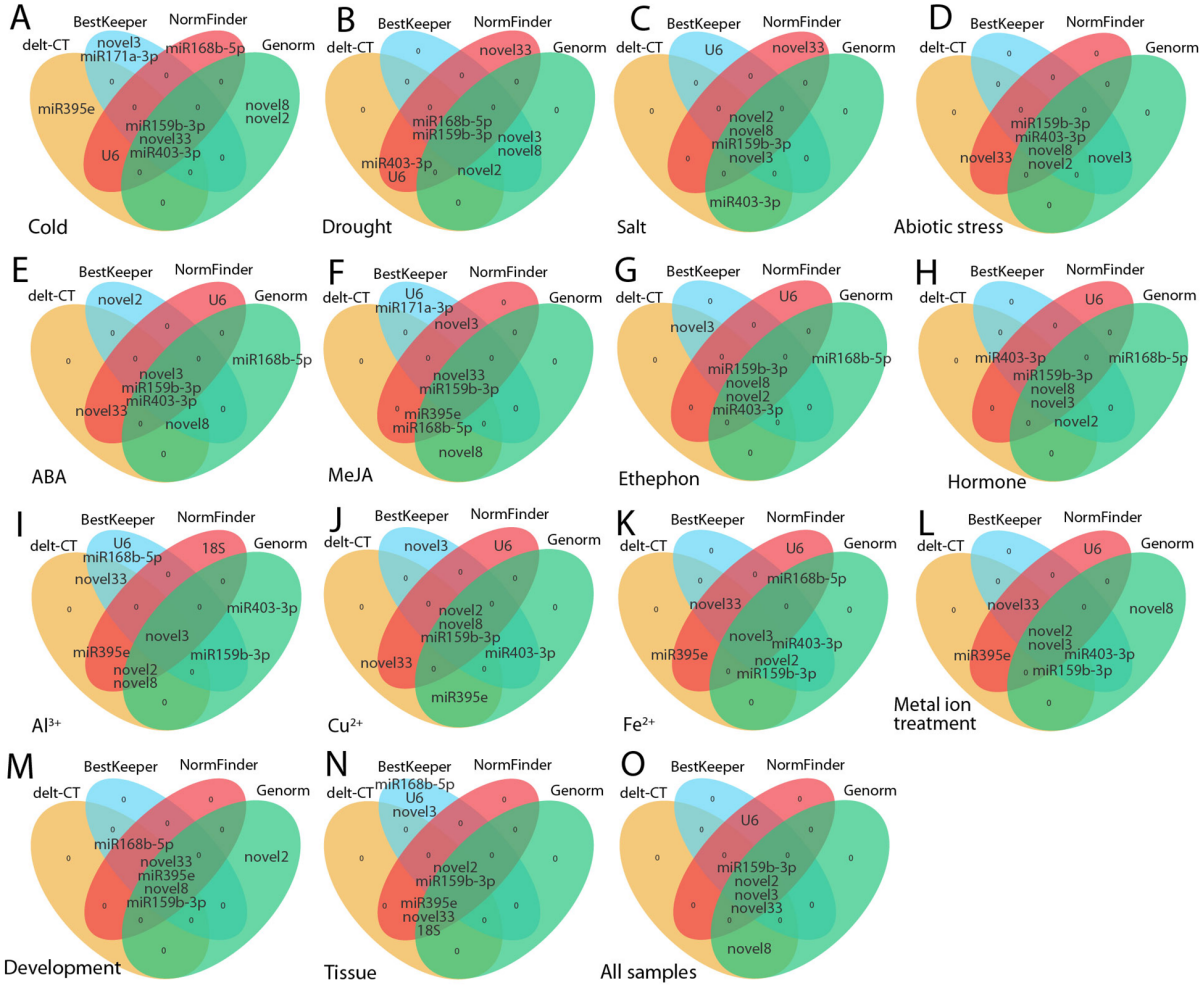
The five most stable internal RGs selected by various programs. The orange, blue, red, and green ovals represent delta-Ct, BestKeeper, NormFinder, and geNorm, respectively. **(A)** Cold, **(B)** drought, **(C)** salt , and **(D)** abiotic stress; **(E)** ABA, **(F)** MeJA, **(G)** ethephon, **(H)** hormone, **(I)** Al3+, **(J)** Cu2+, **(K)** Fe2+, and **(L)** metal ion treatments; **(M)** flowering stages; **(N)** different tissues; and **(O)** across all samples.

This study employed multiple analytical methods (delta-Ct, geNorm, NormFinder, BestKeeper) to assess the stability of candidate RGs. Overall, the top five genes identified by these different algorithms were generally consistent (**Figure 9**). For example, under conditions such as salt stress, abiotic stress, ethephon treatment, flower opening and senescence, and across all samples, at least four genes were consistently identified as stable (**Figures 9C, D, G, M, O**). However, discrepancies were observed among methods. For instance, under cold stress, delta-Ct identified ofr-miR159b-3p and novel33 as the most stable, while BestKeeper and geNorm found ofr-miR159b-3p and ofr-miR403-3p to be the most stable, and NormFinder selected *U6* and ofr-miR159b-3p as optimal (**Figure 9A**). Such variations have also been noted in other plant RG screenings (Vandesompele et al., 2002; Zhang et al., 2018, 2021, d; Wang et al., 2024), likely due to differences in the criteria used for stability evaluation among methods. Therefore, it is important to consider results from multiple algorithms when selecting the most appropriate RG. Subsequently, using ReFinder and geometric means, and based on geNorm, we identified the optimal RG combinations for various conditions as follows: ethephon treatment: novel33 + ofr-miR159b-3p; ABA treatment: novel3 + ofr-miR159b-3p; MeJA treatment: ofr-miR159b-3p + novel8 + novel2 + novel3; hormone treatments: ofr-miR159b-3p + novel8 + novel3; PEG treatment: ofr-miR168b-5p + ofr-miR159b-3p + novel2 (or novel8); salt stress: novel8 + novel3; cold stress: ofr-miR159b-3p + ofr-miR403-3p; abiotic stress: ofr-miR159b-3p + novel8 + ofr-miR403-3p + novel2; Fe²^+^ treatment: novel3 + novel33 + ofr-miR159b-3p; Al³^+^ treatment: novel3 + ofr-miR395e + novel33; Cu²^+^ treatment: novel2 + novel8 + ofr-miR159b-3p; metal ion stress: novel3 + ofr-miR159b-3p + novel2 + novel33 + ofr-miR395e; various tissues: novel2 + ofr-miR395e; flowering stages: novel33 + ofr-miR395e; and all samples: ofr-miR159b-3p + novel3 + novel2 + novel33 + novel8 + *U6* + ofr-miR403-3p + ofr-miR395e + ofr-miR168b-5p (**Table 2**; **Supplementary Table S3**). The miRNAs demonstrated high expression stability under different environmental stresses and developmental stages (**Table 2**; **Supplementary Table S3**), which is consistent with findings from previous studies in other species. Reference miRNAs in plant species have shown greater stability compared to commonly used genes such as *18S* and *ACT* (Zhang et al., 2021c, d). For example, novel16, cln-miR6725, novel1, and *U6* were identified as the most stable RGs for studying miRNA expression in *C. fortunei* under abiotic stresses and hormone treatments (Zhang et al., 2021c). This stability suggests that miRNAs are potential RGs for miRNA expression analysis. Therefore, employing multiple stable reference miRNAs for combined normalization in *O. fragrans* miRNA expression analysis may yield more reliable results. Moreover, the correlation between different stress-related reference miRNAs depends on their expression patterns and regulatory roles in different stress conditions. miRNAs are crucial regulators of gene expression in plants, targeting specific mRNAs to modulate stress responses (Achkar et al., 2016; Michlewski and Cáceres, 2018; Millar, 2020). Consequently, certain reference miRNAs may exhibit stable expression under specific stress conditions, indicating their potential co-regulatory functions or overlapping roles. For example, novel16 is a stable reference in *C. fortunei* under high temperature, cold, drought, and salt stresses (Zhang et al., 2021c), suggesting that these miRNAs may work together through similar regulatory mechanisms, such as controlling specific gene expression pathways (Ferdous et al., 2015). On the other hand, some miRNAs may show stable expression only under certain stress conditions, highlighting their distinct regulatory specificity (Zhang et al., 2017; Luo et al., 2018). Therefore, the correlation between reference miRNAs under different stress conditions is likely shaped by the overlap in the gene networks and signaling pathways they regulate. Further research, especially using high-throughput sequencing, will help elucidate the potential correlations among these miRNAs and deepen our understanding of their synergistic or independent roles in plant stress responses.

Overall, ofr-miR159b-3p, ofr-miR403-3p, ofr-miR395e, ofr-miR168b-5p, novel3, novel2, novel33, novel8, and *U6* were identified as stable RGs under various experimental conditions in *O. fragrans* (**Table 2**; **Supplementary Table S3**). These findings align with previous studies, such as the identification of lpu-miR159a as the most stable RG during somatic embryogenesis in *Lilium pumilum* (Zhang et al., 2017). Additionally, *U6* snRNA has been reported as the most suitable RG under salt and cold stress in grapevine (Luo et al., 2018), whereas miR168 has been shown to perform best under drought stress in grapevine (Luo et al., 2018), and has also been validated for expression analysis in barley (*Hordeum vulgare*) (Ferdous et al., 2015). Notably, the novel miRNAs (novel3, novel2, novel33, and novel8) are rarely reported as RGs in plants (**Table 2**; **Supplementary Table S3**). Despite the limited reports of these novel miRNAs as RGs in plants (Luo et al., 2018; Zhang et al., 2021c), their stability suggests their importance in *O. fragrans*. Exploring their potential functions not only contributes to understanding the mechanisms of miRNA-mediated gene expression regulation in plants, but may also provide novel targets for plant genetic engineering.

Although no completely stable RGs were identified across all experimental conditions, accurate selection of RGs remains crucial for reliable miRNA expression analysis. This study validated the expression levels of ofr-miR166e-5p and ofr-miR396b-3p using both the most stable and least stable RGs, revealing significant impacts on the expression levels and trends of these target genes (**Figures 7**, **8**). These findings underscore the necessity of careful RG selection to ensure accurate gene expression analysis. While this study is the first to systematically evaluate RGs for miRNA expression in *O. fragrans*, it does have some limitations. The research focused on a limited range of abiotic stresses, hormone treatments, and metal ion stresses. Future studies could expand to include additional environmental factors, such as UV radiation and oxidative stress. Furthermore, although qRT-PCR is highly sensitive and specific, it cannot entirely eliminate systemic errors in the experimental process. Future studies could integrate RNA-seq and other high-throughput technologies to provide a more comprehensive and precise analysis of miRNA expression in *O. fragrans*.

## 5 Conclusions

This study represents the first systematic screening and validation of stable RGs for miRNA expression in *O. fragrans*. The results indicate that the optimal RGs vary across different experimental conditions. Specifically, for hormone treatments, ofr-miR159b-3p, novel8, and novel3 exhibited high expression stability; under abiotic stress conditions, ofr-miR159b-3p, novel8, ofr-miR403-3p, and novel2 demonstrated strong stability; in metal ion treatments, novel3, ofr-miR159b-3p, novel33, novel2, and ofr-miR395e were identified as stable genes; across various tissues, novel2 and ofr-miR395e were stable RGs; whereas during flowering stages, novel33 and ofr-miR395e exhibited stable expression. These findings enhance our understanding of the molecular response mechanisms in *O. fragrans* under various stress conditions and provide a scientific basis for breeding and cultivating more resilient varieties. Given the escalating challenges posed by global climate change and environmental pollution, further research into the regulatory mechanisms of *O. fragrans* miRNAs is essential for improving its stress resistance and expanding its applications.

## Data Availability

The original contributions presented in the study are included in the article/**Supplementary Material**. Further inquiries can be directed to the corresponding author/s.
