# Supplementary material for: Validation of suitable reference microRNAs for qRT-PCR in Osmanthus fragrans under abiotic stress, hormone and metal ion treatments
Source: Front Plant Sci. 2025 Feb 14;16:1517225. doi: 10.3389/fpls.2025.1517225 (PMC11868269; doi:10.3389/fpls.2025.1517225)
Supplement: Supplementary file 1 [file Table1.docx]

Supplementary Material

# Supplementary Tables

**Supplementary Table S1.** The expression levels of miRNAs.

|  | S1 | S2 | S3 | S4 | S5 | S6 |
| --- | --- | --- | --- | --- | --- | --- |
| ofr-miR159b-3p | 176,741.41 | 166,144.47 | 124,625.08 | 187,642.16 | 167,616.05 | 133,106.01 |
| ofr-miR168b-5p | 91,604.18 | 88,692.83 | 90,091.66 | 93,418.81 | 66,936.64 | 104,129.50 |
| ofr-miR171a-3p | 20,320.55 | 23,450.51 | 22,858.99 | 15,481.81 | 17,389.13 | 17,950.27 |
| ofr-miR395e | 298.54 | 214.79 | 239.79 | 235.40 | 197.87 | 213.80 |
| ofr-miR403-3p | 3002.98 | 2837.29 | 2797.90 | 2697.28 | 1992.76 | 2304.55 |
| novel2 | 64,873.75 | 77,270.31 | 56,049.40 | 51,658.29 | 58,308.87 | 56,973.99 |
| novel3 | 51,120.81 | 45,608.86 | 50,765.61 | 49,165.40 | 39,074.52 | 38,170.01 |
| novel8 | 15,349.30 | 16,654.39 | 12,080.74 | 12,314.74 | 14,606.24 | 16,542.93 |
| novel33 | 369.17 | 474.24 | 473.67 | 324.01 | 351.38 | 468.72 |

**Supplementary Table S2.** The two most unstable genes screened under different experimental conditions.

|  | delta-Ct | BestKeeper | NormFinder | geNorm |
| --- | --- | --- | --- | --- |
| ethephon treatment | *UBQ4* | *ACT11* | *UBQ4* | *TUA5* |
|  | *ACT11* | *TUA5* | *ACT11* | *ACT11* |
| ABA treatment | *ACT11* | *TUA5* | *ACT11* | *ACT11* |
|  | *TUA5* | *ACT11* | *TUA5* | *TUA5* |
| MeJA treatment | novel33 | *ACT11* | novel33 | *UBQ4* |
|  | *ACT11* | *UBQ4* | *ACT11* | *ACT11* |
| hormone treatment | *ACT11* | *TUA5* | *ACT11* | *ACT11* |
|  | *UBQ4* | *ACT11* | *UBQ4* | *UBQ4* |
| PEG treatment | *TUA5* | *ACT11* | *TUA5* | *TUA5* |
|  | *UBQ4* | *UBQ4* | *UBQ4* | *UBQ4* |
| salt stress | miR171a-3p | *UBQ4* | miR171a-3p | *UBQ4* |
|  | *TUA5* | *TUA5* | *TUA5* | *TUA5* |
| cold stress | *ACT11* | *ACT11* | *ACT11* | *ACT11* |
|  | *TUA5* | *TUA5* | *TUA5* | *TUA5* |
| abiotic stress | *UBQ4* | *UBQ4* | *UBQ4* | *UBQ4* |
|  | *TUA5* | *TUA5* | *TUA5* | *TUA5* |
| Fe^2+^ treatment | *ACT11* | *ACT11* | *ACT11* | *ACT11* |
|  | *TUA5* | *TUA5* | *TUA5* | *TUA5* |
| Al^3+^ treatment | *ACT11* | *ACT11* | *ACT11* | *ACT11* |
|  | *TUA5* | *TUA5* | *TUA5* | *TUA5* |
| Cu^2+^ treatment | *TUA5* | *UBQ4* | *TUA5* | *18S* |
|  | miR168b-5p | *TUA5* | miR168b-5p | miR168b-5p |
| metal ion treatment | *ACT11* | *UBQ4* | miR168b-5p | *ACT11* |
|  | *TUA5* | *TUA5* | *TUA5* | *TUA5* |
| various tissue | miR171a-3p | novel8 | miR171a-3p | *U6* |
|  | miR168b-5p | *TUA5* | miR168b-5p | miR168b-5p |
| flowering stages | *ACT11* | *ACT11* | *ACT11* | *ACT11* |
|  | miR171a-3p | miR171a-3p | miR171a-3p | miR171a-3p |
| all samples | *ACT11* | *ACT11* | *ACT11* | *ACT11* |
|  | *TUA5* | *TUA5* | *TUA5* | *TUA5* |

**Supplementary Table S3.** Comprehensive ranking of stability of candidate RGs.

|  | cold stress | salt stress | PEG treatment | abiotic stress | ABA treatment | MeJA treatment | ethephon treatment | hormone treatment |
| --- | --- | --- | --- | --- | --- | --- | --- | --- |
| 1 | miR159b-3p | novel3 | miR168b-5p | miR159b-3p | novel3 | miR159b-3p | novel33 | miR159b-3p |
| 2 | miR403-3p | novel8 | novel8 | novel8 | miR159b-3p | novel8 | miR159b-3p | novel8 |
| 3 | novel33 | novel2 | miR159b-3p | miR403-3p | miR403-3p | novel2 | miR395e | novel3 |
| 4 | *U6* | miR159b-3p | novel2 | novel2 | novel33 | novel3 | novel3 | novel2 |
| 5 | miR395e | miR403-3p | novel3 | novel3 | novel8 | miR403-3p | miR168b-5p | miR403-3p |
| 6 | novel3 | novel33 | miR403-3p | novel33 | *U6* | *U6* | *U6* | *U6* |
| 7 | novel8 | *U6* | *U6* | miR168b-5p | novel2 | miR168b-5p | novel8 | miR168b-5p |
| 8 | miR168b-5p | *18S* | novel33 | *U6* | miR168b-5p | miR395e | miR171a-3p | miR395e |
| 9 | novel2 | miR395e | miR395e | miR395e | miR171a-3p | miR171a-3p | novel2 | miR171a-3p |
| 10 | miR171a-3p | miR168b-5p | miR171a-3p | miR171a-3p | miR395e | *18S* | miR403-3p | novel33 |
| 11 | *18S* | *ACT11* | *18S* | *18S* | *18S* | *TUA5* | *18S* | *18S* |
| 12 | *UBQ4* | miR171a-3p | *ACT11* | *ACT11* | *UBQ4* | *novel33* | *UBQ4* | *TUA5* |
| 13 | *ACT11* | *UBQ4* | *TUA5* | *UBQ4* | *ACT11* | *UBQ4* | *TUA5* | *ACT11* |
| 14 | *TUA5* | *TUA5* | *UBQ4* | *TUA5* | *TUA5* | *ACT11* | *ACT11* | *UBQ4* |
|  | Al^3+^ treatment | Cu^2+^ treatment | Fe^2+^ treatment | metal ion treatment | various tissues | flowering stages | all samples |  |
| 1 | novel3 | novel2 | novel33 | novel3 | novel2 | novel33 | miR159b-3p |  |
| 2 | miR395e | novel8 | novel3 | miR159b-3p | miR395e | miR395e | novel3 |  |
| 3 | novel33 | miR159b-3p | miR159b-3p | novel33 | miR159b-3p | novel8 | novel2 |  |
| 4 | miR403-3p | miR403-3p | miR403-3p | novel2 | novel33 | miR168b-5p | novel33 |  |
| 5 | novel2 | novel3 | miR395e | miR395e | *18S* | novel2 | novel8 |  |
| 6 | miR159b-3p | miR395e | novel2 | miR403-3p | novel3 | miR159b-3p | *U6* |  |
| 7 | novel8 | novel33 | *U6* | novel8 | *UBQ4* | *U6* | miR403-3p |  |
| 8 | *U6* | *U6* | miR168b-5p | *U6* | miR168b-5p | *TUA5* | miR395e |  |
| 9 | *18S* | miR171a-3p | miR171a-3p | miR171a-3p | *U6* | miR403-3p | miR168b-5p |  |
| 10 | miR168b-5p | *ACT11* | *18S* | *18S* | *ACT11* | novel3 | *18S* |  |
| 11 | miR171a-3p | *UBQ4* | novel8 | miR168b-5p | miR403-3p | *18S* | *miR171a-3p* |  |
| 12 | *UBQ4* | *18S* | *UBQ4* | *UBQ4* | novel8 | *UBQ4* | *UBQ4* |  |
| 13 | *ACT11* | *TUA5* | *ACT11* | *ACT11* | *TUA5* | *ACT11* | *ACT11* |  |
| 14 | *TUA5* | miR168b-5p | *TUA5* | *TUA5* | miR171a-3p | miR171a-3p | *TUA5* |  |
